# A new combination technique of the modified Robicsek wire fixation and plate fixation achieves effective repair of transverse sternal fracture: a report of two cases

**DOI:** 10.1186/s40792-021-01267-8

**Published:** 2021-08-12

**Authors:** Yoshifumi Fuke, Tomoki Ushijima, Sho Matsuyama, Satoshi Kimura, Hiromichi Sonoda, Sayaka Osawa, Jun Maki, Kentaro Tokuda, Kenta Momii, Akira Shiose

**Affiliations:** Department of Cardiovascular Surgery, Emergency and Critical Care Center, and Intensive Care Unit, Kyushu University Hospital, 3-1-1 Maidashi, Higashi-ku, Fukuoka, 812-8583 Japan

**Keywords:** Sternal fracture, Flail chest, Modified Robicsek technique, Plate fixation

## Abstract

**Background:**

To maximize the therapeutic effect for complicated sternal fracture, we should know advantages and disadvantages of each surgical repositioning technique, and the choice of an appropriate procedure is essential. We report two successful cases for which a combination of two existing techniques, modified Robicsek wire fixation and locked titanium plate fixation, was applied to transverse sternal fracture with flail chest.

**Case presentation:**

One patient experienced a transverse sternal and rib fracture due to a traffic injury. Flail chest due to a highly displaced transverse sternal fracture made withdrawal of the ventilator impossible. Another patient, who developed fulminant myocarditis, experienced a transverse sternal fracture resulting from chest compression during cardiopulmonary resuscitation. Severe paradoxical respiratory movement was a limiting factor for cardiac and respiratory rehabilitation. In both cases, a transverse sternal fracture was difficult to correct non-invasively and indicated surgical repair. The surgical repositioning and fixation greatly contributed to the improvement of the respiratory movement, and the patients were successfully withdrawn ventilator support.

**Conclusion:**

The combination of modified Robicsek wire fixation and locked titanium plate fixation for a complicated sternal fracture employs the complementary and comparative advantages of each procedure and effective fixation may be achieved.

## Background

Most sternal fractures are treated conservatively; but some, such as the largely dislocated sternum, flail chest, and nonunion joints formation, are difficult to correct non-invasively and require surgical intervention**.** Common surgical repositioning techniques are those in which a wire, a sternum pin, or a plate, are used [1]. To maximize the therapeutic effect for complicated sternal fracture, we should know advantages and disadvantages of each procedure, and the choice of an appropriate surgical procedure is essential. We report two successful cases for which a combination of two existing techniques, modified Robicsek wire fixation [2] and locked titanium plate fixation [3], was applied to transverse sternal fractures associated with flail chest.

## Case presentation

### Case 1

**Fig. 1:**
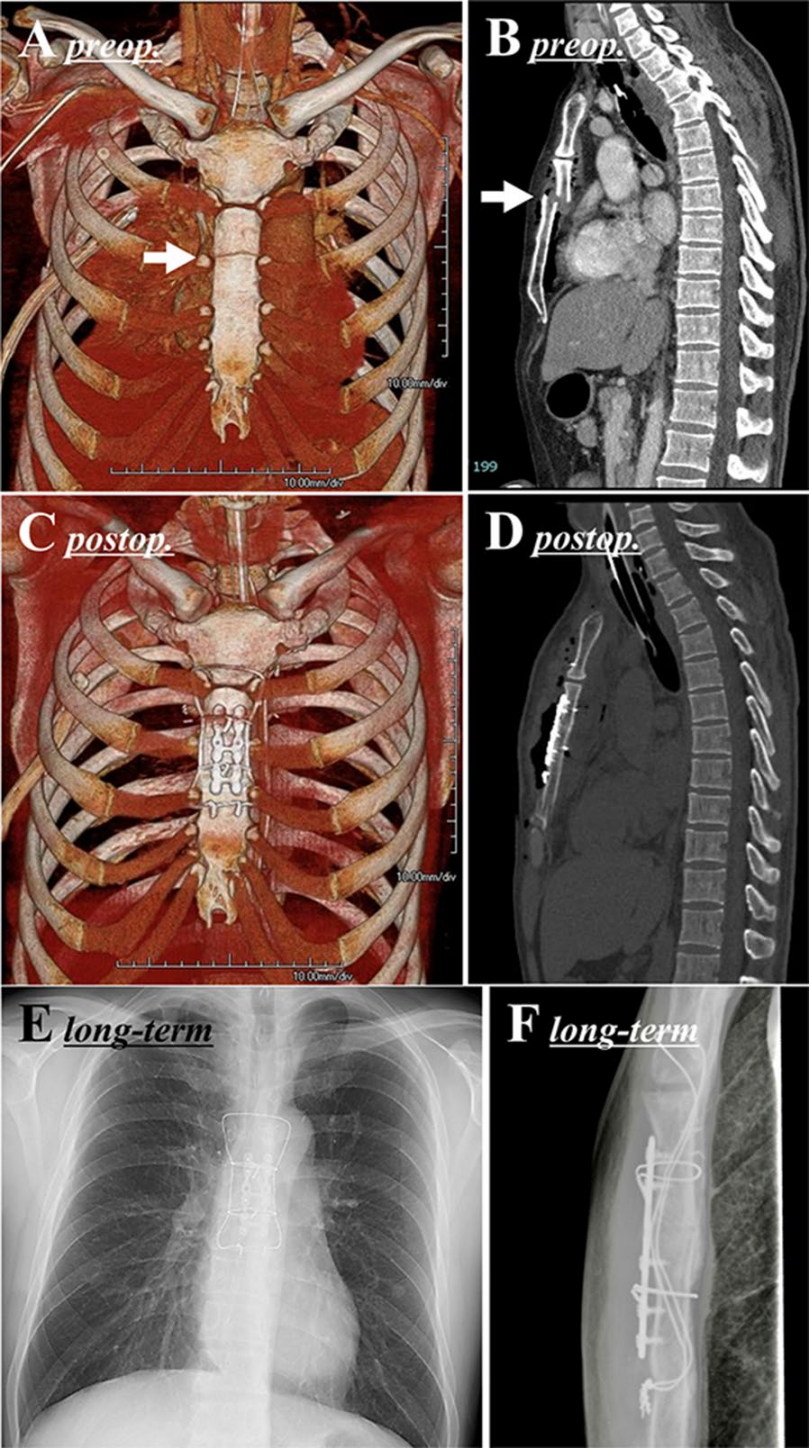
Pre- and post-operative images in Case 1. Preoperative computed tomography (**A**, **B**) demonstrated a fracture line at the level of the 2nd lower intercostal margin (arrow) and the posterior displacement of the upper sternum. Postoperative computed tomography (**C**, **D**) and long-term radiograph (**E**, **F**) confirmed that the sternum was properly reduced

A 40-year-old man in a traffic accident was transported to our hospital. He was in a state of shock due to multiple trauma. The anatomical severity according to the Injury Severity Score was 26 points; the physiological severity according to the Revised Trauma Score was 7.10; and the probability of survival according to the Trauma and Injury Severity Score calculated from these values was 95.8% [4–6]. Bone computed tomography (CT) showed a highly displaced transverse sternal fracture (Fig. 1A, B) and rib fracture. Paradoxical breathing, i.e., flail chest, became apparent with spontaneous breathing and tachypnea and hypercapnia made withdrawal of the ventilator impossible. A transverse sternal fracture with flail chest indicated surgical repair to withdraw ventilator support.

On the 5th day after the injury, we performed surgical repositioning and fixation of the sternum using the modified Robicsek technique combined with SternaLock® (Zimmer Biomet, Jacksonville, FL, US) plate fixation as described in the “*Operative technique*” section below. There was minimal blood loss. Due to the repositioning and stabilization of the sternal fracture, flail chest entirely disappeared, and the ventilator was successfully removed the day after the operation. A postoperative CT scan confirmed that the sternum was properly repositioned (Fig. 1C, D). Currently, 1.5 years have passed since the operation, and excellent fixation is still maintained (Fig. 1E, F).

### Operative technique (Fig. 2)

**Fig. 2:**
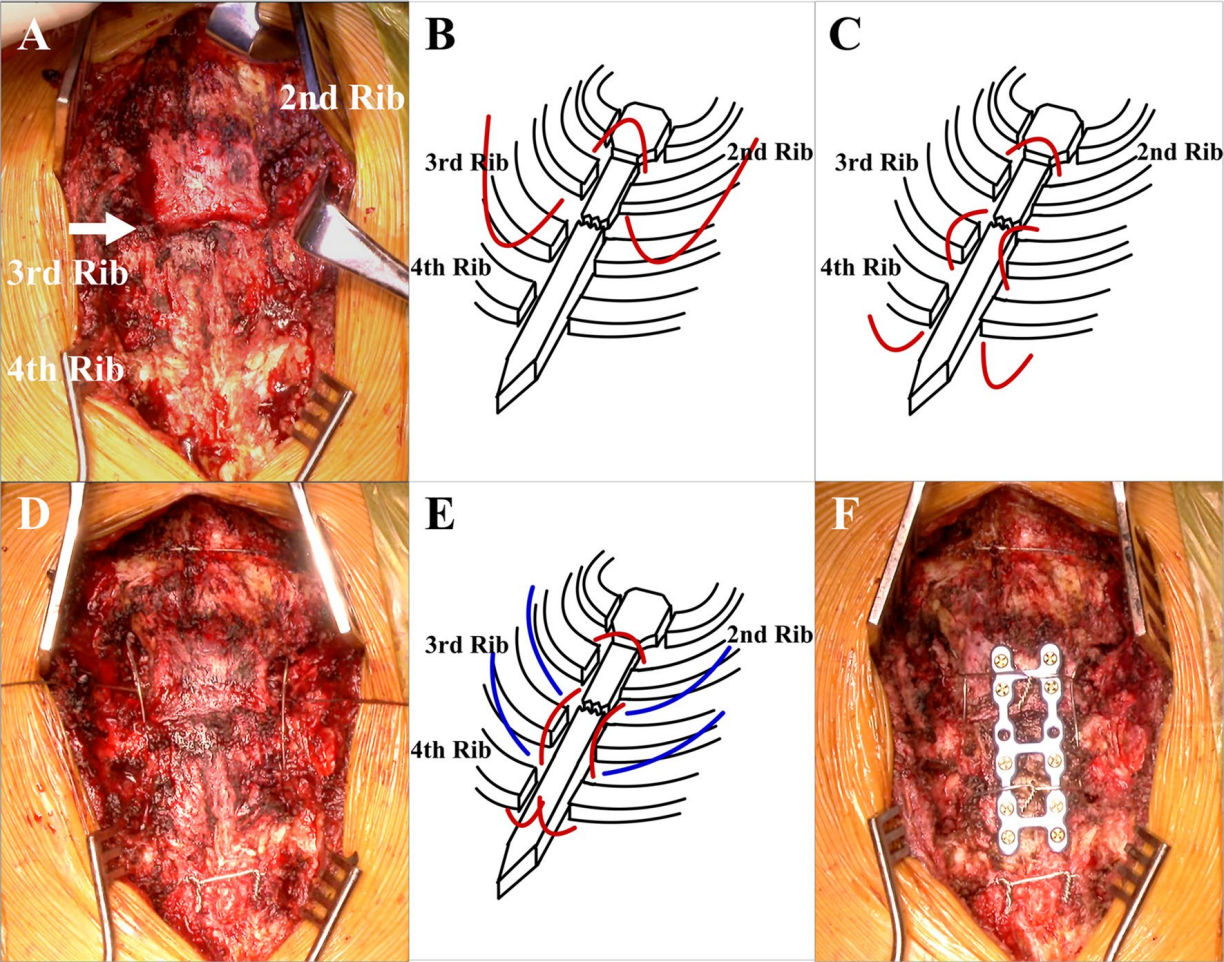
Intraoperative photographs and schemas in Case 1. **A** The front of the sternum was exposed, and a fracture line was found at the 2nd lower intercostal margin (arrow). **B**, **C** According to the modified Robicsek technique with some minor modifications, both ends of a stainless-steel wire were advanced via the 1st intercostal space and drawn out via the 4th intercostal space, alternating from the posterior side of the 2nd rib to the anterior side of the 3rd rib and back to the poster side of the 4th rib. **D**, **E** Stainless-steel wires were passed horizontally at the 2nd and 3rd interscostals. **F** The front of the sternum was fixed with a 12-pin H-shaped SternaLock® plate, and two transverse stainless-steel wires were tied in front of the sternum at the 2nd and 3rd intercostals

A fracture line was found at the level of the 2nd lower intercostal margin (Fig. 2A), and the upper sternum was largely displaced posterior to the lower sternum. The intercostals on both sides were separated at the margin of the sternum. Care was taken so as not to injure the internal thoracic and intercostal arteries and veins, whose locations were confirmed by preoperative CT.

The modified Robicsek technique was performed as described previously [2], with some modifications. One end of a stainless-steel wire was passed between several ribs, across the fracture line, and the other end was passed through the contralateral rib. The ends of the wire were finally tied in front of the sternum. In this case, both ends of a stainless-steel wire were advanced via the 1st intercostal space and drawn out via the 4th intercostal space, alternating from the posterior side of the 2nd rib to the anterior side of the 3rd rib and back to the posterior side of the 4th rib (Fig. 2B, C), and were finally tied in front of the sternum at the 4th intercostal level (Fig. 2D). Subsequently, two stainless-steel wires were passed horizontally at the 2nd and 3rd intercostals (Fig. 2E). Next, the sternum was fixed with a 12-pin H-shaped SternaLock® plate. Last, the transverse wires were tied in front of the sternum (Fig. 2F). The parasternal longitudinal wires acted as struts to the transverse wires.

### Case 2

**Fig. 3:**
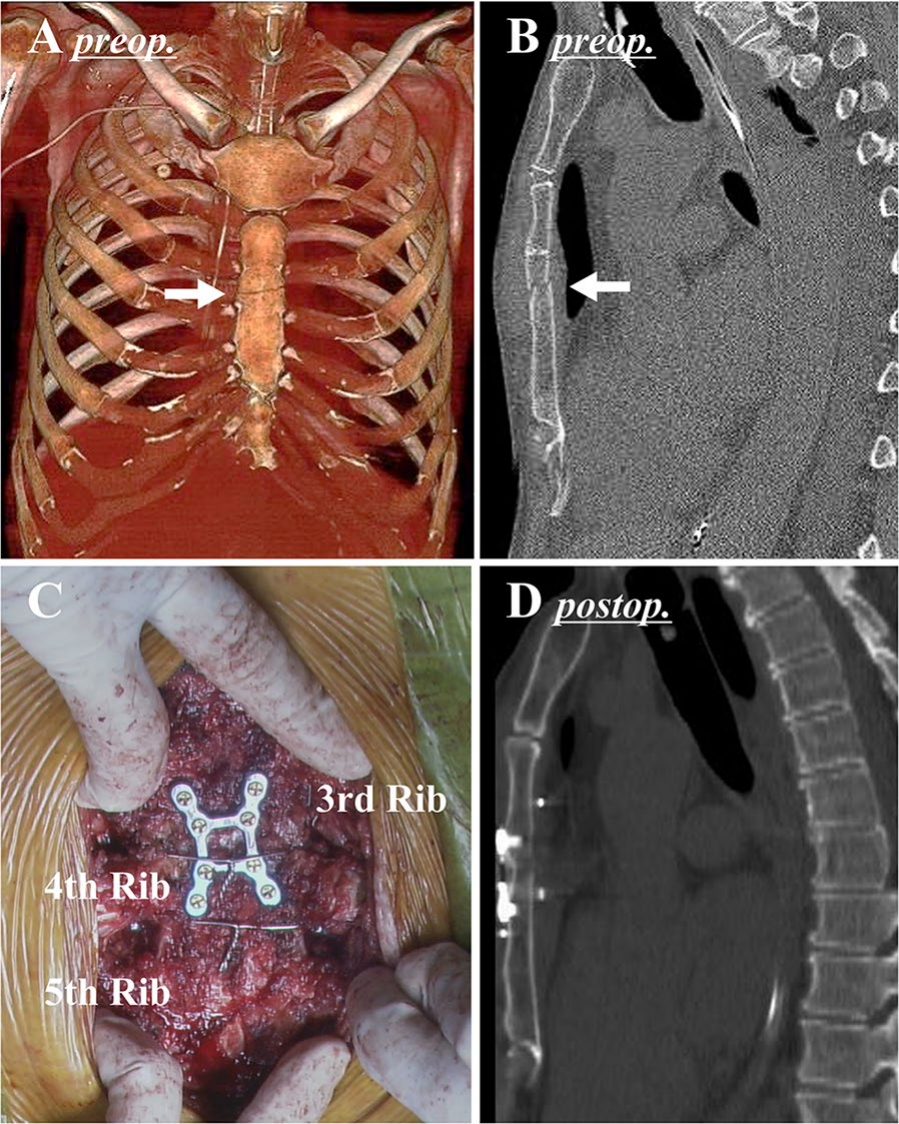
Preoperative image and intraoperative photo in Case 2. **A**, **B** Preoperative computed tomography demonstrated that a fracture line was found at the 3rd intercostal level (arrow) and the posterior displacement of the lower sternum. **C** The sternum was reduced with a combination of modified Robicsek wire fixation and plate fixation using an 8-pin H-shaped SternaLock® plate. **D** Postoperative computed tomography confirmed that the sternum was properly reduced

A 70-year-old man developed rapidly progressive cardiogenic shock due to fulminant myocarditis, and cardiopulmonary support was immediately initiated by Ecpella, a combination of peripheral veno-arterial extracorporeal membrane oxygenation (ECMO) and an Impella® (Abiomed, Danvers, MA, US) percutaneous left ventricular assist device. Those both were successfully discontinued within 2 weeks after the initiation. Bone CT showed a transverse sternal fracture (Fig. 3A, B), resulting from chest compression during cardiopulmonary resuscitation. Although this was not much of an issue during the ECMO support with the less breathing effort, paradoxical respiratory movement became apparent with spontaneous breathing after weaning from ECMO and tachypnea and tachyarrhythmia made it difficult to withdraw ventilator support. Flail chest was a limiting factor for cardiac and respiratory rehabilitation, which indicated surgical repair.

On the 21st day after the injury, the fractured sternum was surgically repaired using the same combination technique as Case 1. Briefly, a combination of the modified Robicsek technique and sternal plating with an 8-pin H-shaped SternaLock® was performed (Fig. 3C). In this case, pseudo-articularization had already started, and the fragments of the exposed sternum were entirely separated after thorough dissection around the fracture line. Although preoperative CT under the condition of internal fixation by ventilator support appeared a simple fracture (Fig. 3A, B), in fact, pseudo-articularization probably resulted in paradoxical respiratory movement. The separated fragments of the sternum were manually repositioned and the combination technique was applied. There was very little bleeding. After surgery, significant improvement of the breathing movement enabled facilitating cardiac and respiratory rehabilitation as heart failure treatment. Finally, we were able to withdraw ventilator support under stable hemodynamics and respiratory conditions. Sternal fixation played a role of severe heart failure treatment for this patient. A postoperative CT scan confirmed that the sternum was properly repositioned (Fig. 3D). Two years after surgery, the patient has now fully recovered and has resumed his normal daily activities.

## Discussion

Sternal plating can prevent misalignment of the sternum, facilitate sternal healing and stability, reduce postoperative pain, and decrease in the long-term nonunion rate. The SternaLock® plating system has mainly been applied for median sternotomy patients who have a high-risk of sternum wound complications. This distinctive H-shaped plate can provide excellent rigidity and contributes to a decrease in multi-directional displacements [1, 3, 7]. However, pinning depends on the state of the bone, e.g., the extent of the fractured surface and the fragile bone cortex. Repositioning and fixation using only a plate may not be suitable for severely dislocated sternums. For such cases, some approximation of the sternal fragments may be necessary prior to sternal plating.

Various wire fixation techniques, to achieve sternal stability, have been introduced, each with its advantages and disadvantages. Repositioning and fixation using only a wire may cause problems such as pseudo-articularization and damage to the brittle fractured sternum [1, 2]. The well-known conventional Robicsek technique is superior to simple wire fixation in terms of sternal stability and prevention of rupture of the sternum, but its disadvantage is that blood supply to the sternum can be disrupted due to wire strangulation around the costal cartilage. The modified Robicsek technique, however, does not strangulate costal cartilage while maintaining stability of the sternum, and the spared intercostal arteries and veins may contribute to maintaining blood flow to the sternum. The modified Robicsek technique is favorable in terms of prevention of sternum wound complications as well as sternal stability [2].

We applied a new combination of two existing techniques to achieve the benefits of both. Each fixation technique is common procedure, but there are few reports of a combination thereof. When repositioning a severe transverse sternal fracture resulting in paradoxical respiratory movement, the therapeutic effects of plate fixation alone may be insufficient. We believe that, to maximize the therapeutic effects of plate fixation, the modified Robicsek wire fixation offers an approximation of the largely displaced sternum prior to plating, and subsequent plating can stabilize the sternum more effectively. This complementary combination can achieve effective fixation and long-term stability simultaneously.

A transverse sternal fracture causes paradoxical respiratory movement and often makes it difficult to withdraw ventilator support. Although bone CT images are effective for diagnosis, it may be difficult to accurately assess the degree of displacement of the sternum. This is a pitfall in assessing the severity of sternal fracture. A clinically symptomatic transverse sternal fracture can be indicative of surgical intervention.

In addition, this combination technique, which can provide multi-directional sternal stability, has versatility for fixation of the fragile sternum during median sternotomy. This is knowledge that cardiovascular and thoracic surgeons should know.

## Conclusions

In two cases, the combination of the modified Robicsek wire fixation technique and SternaLock® plate fixation was favorable for repositioning of a transversely fractured sternum. This combination technique employs the complementary and comparative advantages of each procedure and effective fixation may be achieved.

## Data Availability

The data supporting the conclusions of this case report are included within the article.

## Abbreviations

CT: Computed tomography
ECMO: Extracorporeal membrane oxygenation
